# Pheniramine Maleate Dependence : A Case Study

**DOI:** 10.1192/bjo.2026.11759

**Published:** 2026-06-30

**Authors:** Iqra Ain Ali, Imtiaz Ahmad Dogar, Maheen Ijaz, Abdul Rehman Asgher, Fatima Tuz Zahra

**Affiliations:** 1 Faisalabad Medical University, Faisalabad, Pakistan; 2 Allama Iqbal Medical College, Lahore, Pakistan

## Abstract

**Aims:**

Addiction to over-the-counter (OTC) drugs is a poorly acknowledged clinical issue, especially in developed and developing nations when the regulatory supervision is less strict. Pheniramine hydrogen maleate (PHM) is an antihistamine of the first generation that is broadly used in allergic problems and can be freely bought without a prescription as Avil. Because of its sedative, anxiolytic, and euphoric effects, there is the possibility of misuse of pheniramine. Despite the description of psychological tolerance as well as withdrawal symptoms, there are very few reported cases of pheniramine dependence in the psychiatric literature. The case under consideration is a longitudinal dependence on pheniramine, which accentuates its clinical progression and withdrawal symptoms and explains the necessity of clinicians being aware of the abuse of the widely popular antihistamines.

**Methods:**

A 35-year-old married businessman presented with symptoms of substance withdrawal such as restlessness, irritability, vomiting, diarrhea, headache, and insomnia following cessation of chronic pheniramine use. His past history of intravenous pheniramine (Avil) use dates back to 12 years, when he was taking 4–6 vials per day and at times 10 vials. He began using it when he was prescribed injectable pheniramine and dexamethasone to treat a chronic cough and body aches. He continued to self-administer even though he underwent routine investigations because he believed that he had recovered. He had several unsuccessful attempts at discontinuation. He had two years of abstinence after inpatient detoxification and asthma treatment but relapsed due to accidental exposure to pheniramine during his hospitalization. The mental status examination presented guilt, health preoccupation, and decreased attention. The ICD-10 criteria were used to diagnose Psychoactive Substance Dependence Syndrome (pheniramine) and comorbid bronchial asthma. He was in an action stage of the change model.

**Results:**

Pheniramine is also associated with the abuse potential due to its sedation and anticholinergic effects, specifically because of its use as an intravenous method of administration that enhances the bioavailability and the reinforcing effects. The likelihood of dependence emerged due to repeated self-medication of PHM for respiratory symptoms. The unexpected reexposure emphasized the importance of paying attention to medication history and pharmacovigilance.

**Conclusion:**

Although rare, dependency on pheniramine is a severe and underestimated clinical issue related to OTC medications. This example highlights that combined multidisciplinary management, more stringent control over the sale of antihistamine products, and increased awareness of clinicians are required. Early psychiatric intervention, structured detoxification, and psychosocial support are essential in order to recover in the long term and prevent relapse.

